# Supplementary material for: Belief state representation in the dopamine system
Source: Nat Commun. 2018 May 14;9:1891. doi: 10.1038/s41467-018-04397-0 (PMC5951832; doi:10.1038/s41467-018-04397-0)
Supplement: Supplementary file 1 — Supplementary Information [file 41467_2018_4397_MOESM1_ESM.pdf]

Supplementary Information

Belief State Representation in the Dopamine System

Benedicte M. Babayan, Naoshige Uchida and Samuel J. Gershman

Supplementary Figures

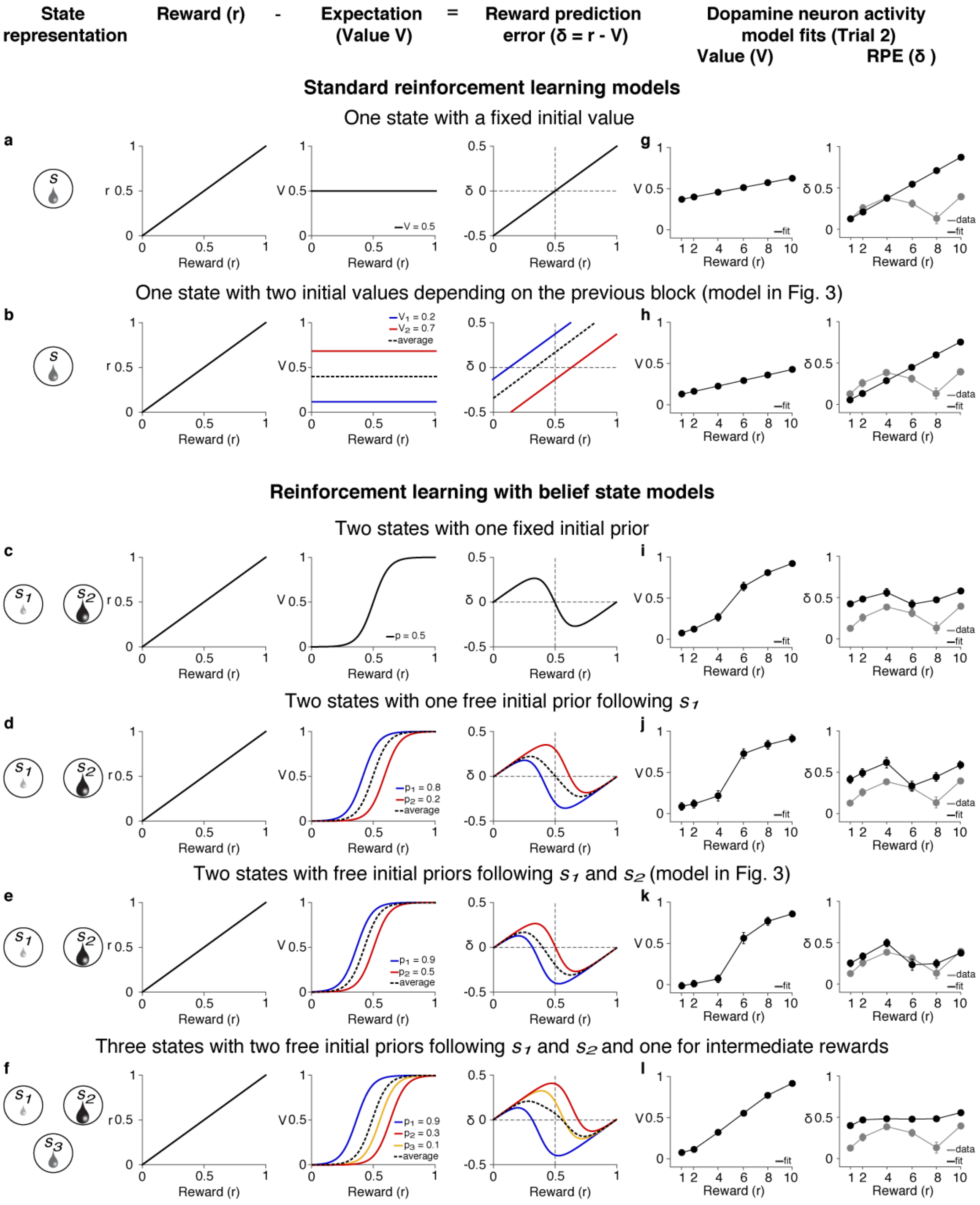

Supplementary Figure 1. RL models tested. Six model variants were tested. **a-f** For each model, from left to right, the model's state space is represented, followed by the delivered reward (r), which is compared to the expectation (value V of the state or belief

state) to compute the RPE (δ). The 4th column shows the theoretical RPE, which is centered around 0. The main distinction between the standard RL models (**a, b**) and the belief state models (**c**-**f**) is the state representation, with a single state in the case of the standard RL model due to the ambiguity of the odor. **g-l** The last two columns show the theoretical value and RPE on trial 2, obtained by fitting each model's RPE to the GCaMP responses (see parameters in Supplementary Table 1) using linear regression. This regression accounted for the fact that in our task most reward responses were positive, likely due to temporal uncertainty[1,2]. Only using belief states allows reproducing the non-monotonic pattern of dopamine RPEs observed on trial 2. **a** Standard RL with a fixed initial value for the state at 0.5 (V, averaged between the trained states s_1 and s_2), leading to a monotonically increasing RPE. **b** Variant of the standard RL model with free initial values for the state depending on the previous block, following s_1 (value V_1) and s_2 (value V_2). The averaged value is indicated by a black doted line. This also leads to a monotonically increasing RPE, only the intercept is affected. **c** RL with belief state using a fixed initial prior for all states' likelihood at 0.5 (p). The value of the belief state depends on the reward size, with smaller rewards being more likely to being similar to s_1, resulting in a low value, and with bigger rewards being more likely to being similar to s_2, resulting in a high value. This expectation function predicts a non-monotonic pattern in RPEs when compared to the delivered reward. **d** Variant of the RL with belief state using a free initial prior following s_1 (prior p_1), constraining both priors to sum to 1 - notice the averaged prior in black dotted line identical to the prior in **c**. **e** Variant of the RL with belief state using two free initial priors following s_1 (prior p_1) and following s_2 (prior p_2). Notice how this allows the averaged prior function in black dotted line to be biased towards being in s_2 in this example, leading to an asymmetric non-monotonic pattern of prediction error. **f** Variant of the RL with belief state using three states, one additional one for intermediate rewards (s_3), and three initial free priors following s_1 (prior p_1), following s_2 (prior p_2) and for intermediate rewards (prior p_3).

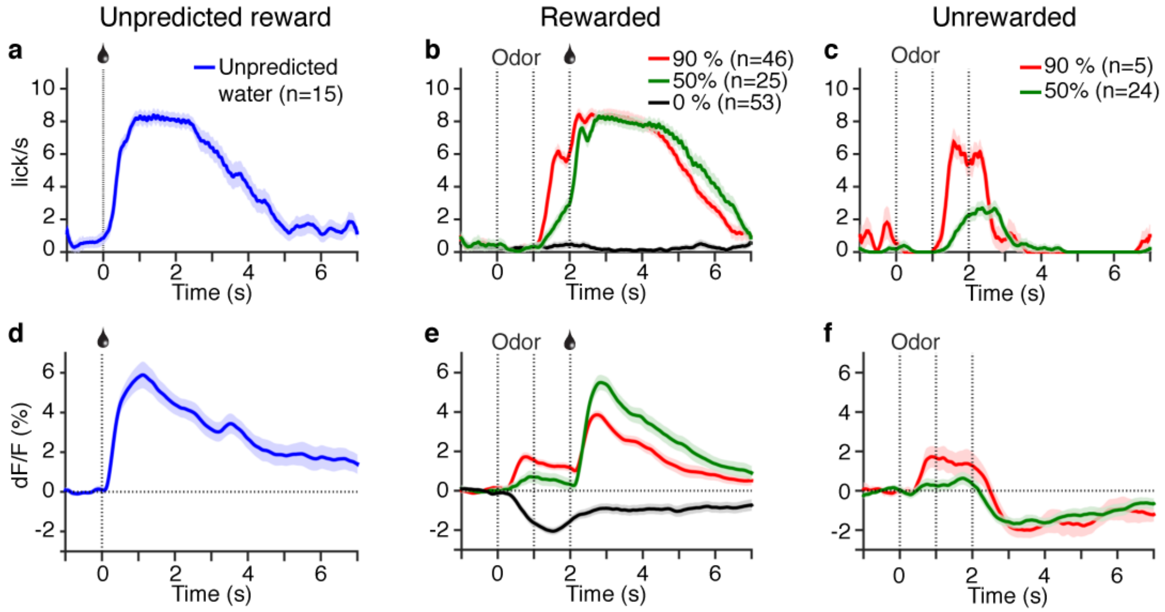

Supplementary Figure 2. Behavior and fibre photometry recordings of VTA dopamine neurons in classical conditioning. We presented 3 odors, which predicted the delivery of water one second later with either 90% (red), 50% (green) or 0% (black) probability. Unpredicted water was delivered on 10% of trials. **a** On unpredicted water delivery trials, the mouse licked on water delivery. **b**, **c** For odors predicting reward with 90% or 50% probability, the mouse showed anticipatory licking after odor presentation proportional to the probability of reward delivery. **d**-**f** The activity of dopamine neurons on reward delivery showed a canonical RPE pattern: strongest response to fully unpredicted reward (**d**), decreased responses to predicted rewards (**e**) and dip at reward omission (**f**). Dopamine neurons activity at cue onset was proportional to the value of the cue (**e**, **f**). Data represents mean ± s.e.m. n indicates number of trials.

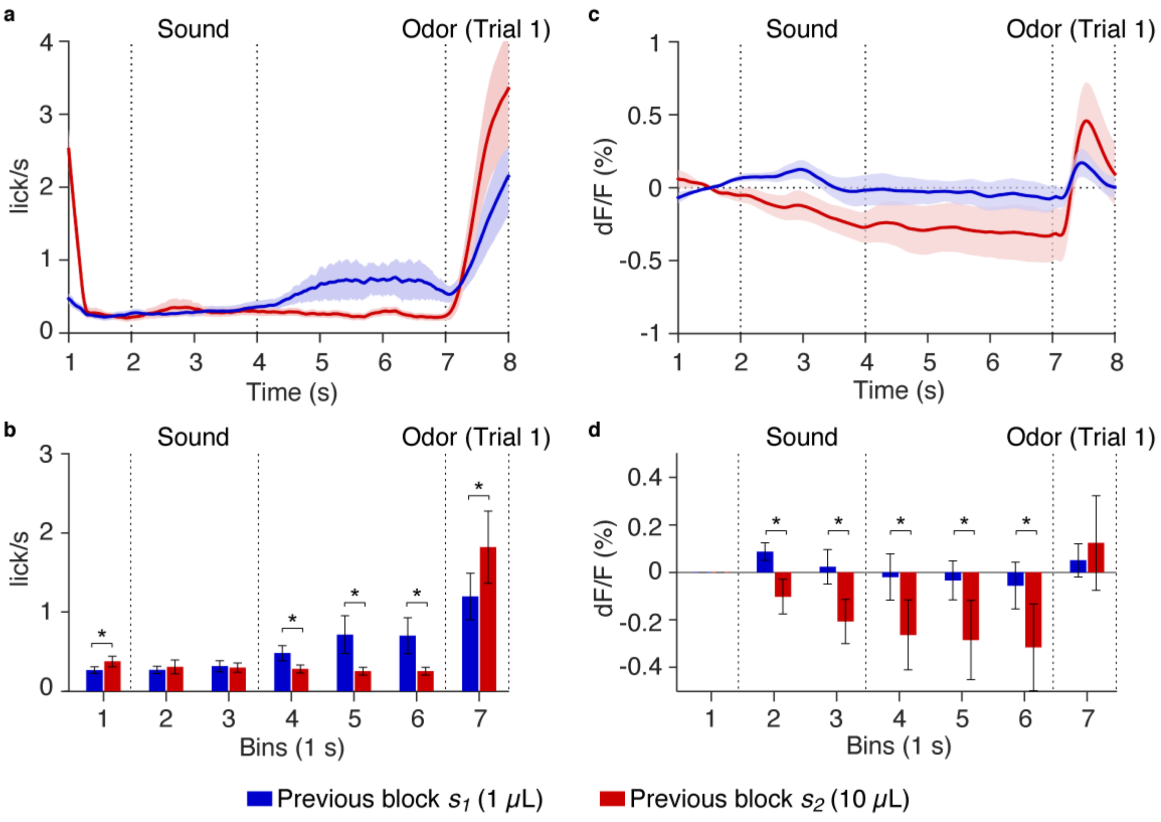

Supplementary Figure 3. Anticipatory licking and dopamine activity at block start. **a** Anticipatory licking at block start. **b** Anticipatory licking quantified over 1 second bins. **c** GCaMP activity at block start. **d** GCaMP activity quantified over 1 second bins. Data is separated based on the previous block (s_1 and s_2). The sound cue signals block start and is followed by the odor cue for trial 1. Data represents mean ± s.e.m. * p > 0.05 for post-hoc paired Wilcoxon tests. n = 11 mice

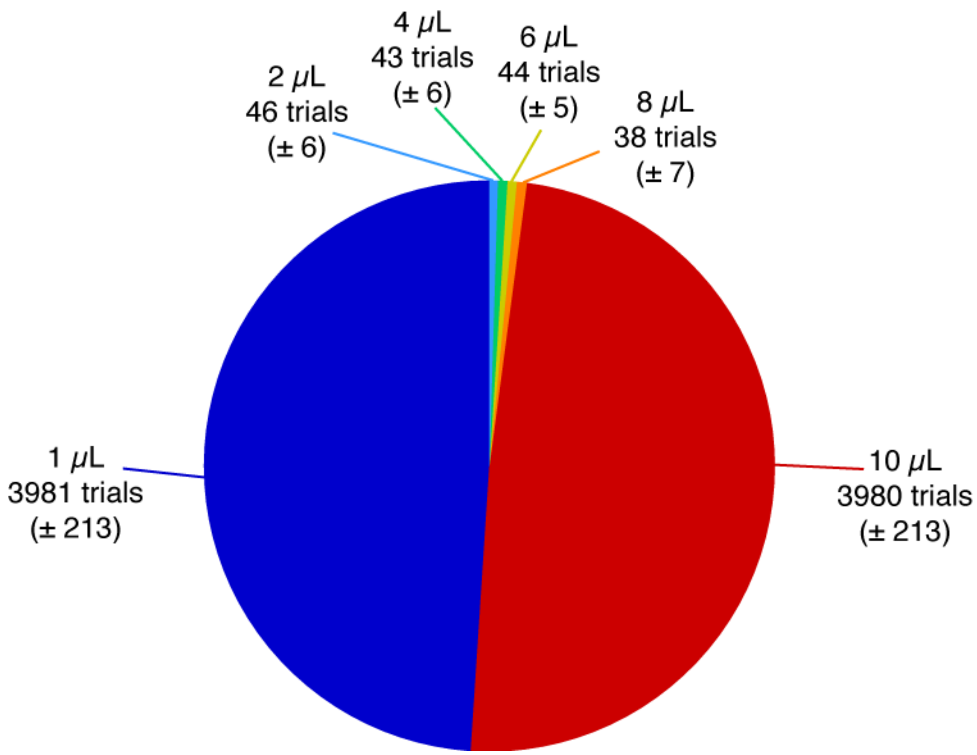

Supplementary Figure 4. Number of trial types mice experienced over the whole training. For each volume, the average number of trials (with s.e.m.) experienced by each mouse is indicated. Each intermediate reward (2 to 8 μL) was experienced less than 0.5 % of the number of training trials each mouse experienced. n = 11 mice.

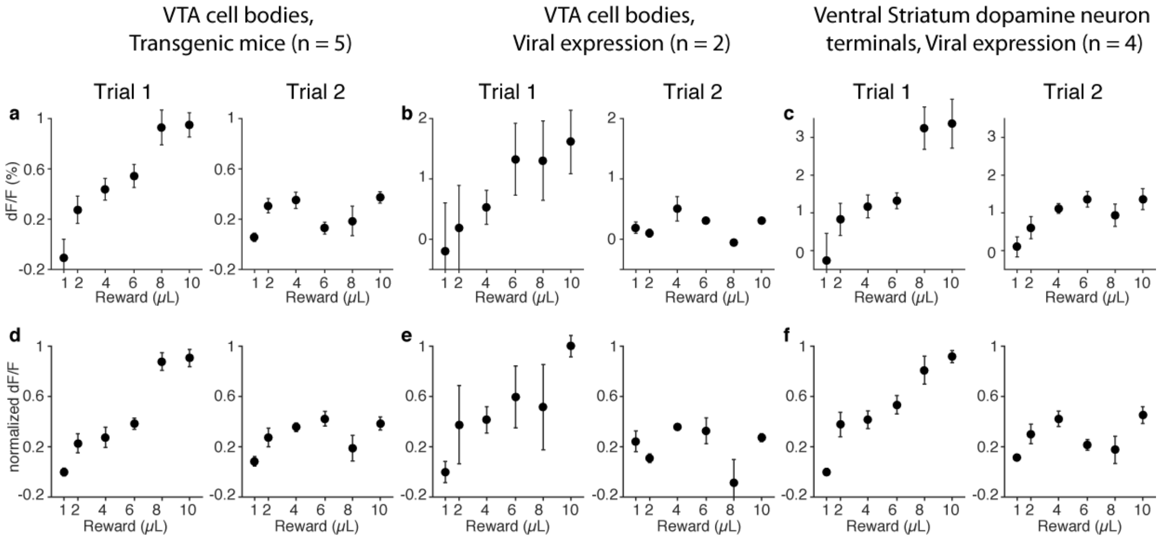

Supplementary Figure 5. Dopamine responses on trials 1 and 2 plotted separately based on recording conditions. **a**, **d** Dopamine responses in mice expressing transgenetically GCaMP6f in DAT-positive neurons and recorded from VTA cell bodies (n = 5). **b**, **e** Dopamine responses in mice expressing GCaMP6f through a viral construct in DAT-positive neurons and recorded from VTA cell bodies (n = 2). **c**, **f** Dopamine responses in mice expressing GCaMP6f through a viral construct in DAT-positive neurons and recorded from dopamine neuron terminals in the ventral striatum (n = 4). The upper row (**a** - **c**) shows the average across mice, while the lower row (**d** - **f**) shows the same average after normalizing within mice through min-max normalization using trial 1's response as reference for the minimum and maximum values. This normalization corrects for the different amplitudes in GCaMP signals across the different recording conditions, but preserves the features observed in each recording condition. Note that the monotonicity and non-monotonicity of the responses in trials 1 and 2, respectively, are observed in each recording condition (**a** - **c**). Data represents mean ± s.e.m.

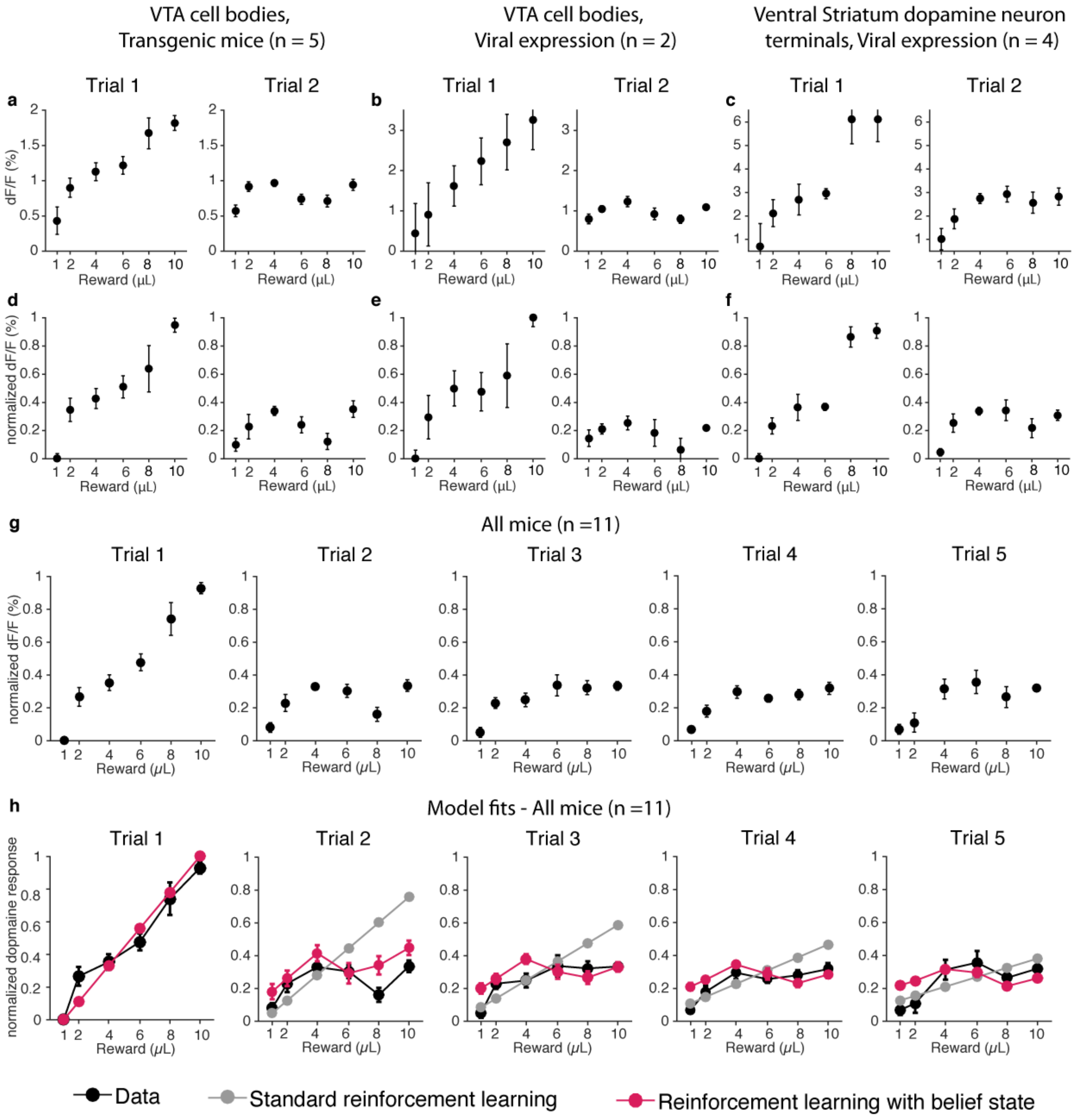

Supplementary Figure 6. Data and model fits on peak dopamine response. Quantifying the peak dF/F response following reward delivery recapitulates the results obtained by quantifying the average response over one second post reward delivery. **a, d** Dopamine responses on trials 1 and 2 for mice expressing transgenetically GCaMP6f in DAT-positive neurons and recorded from VTA cell bodies (n = 5). **b, e** Dopamine responses on trials 1 and 2 for mice expressing GCaMP6f through a viral construct in DAT-positive neurons and recorded from VTA cell bodies (n = 2). **c, f** Dopamine responses on trials 1 and 2 for mice expressing GCaMP6f through a viral construct in DAT-positive neurons and recorded from dopamine neuron terminals in the ventral striatum (n = 4). The upper row (**a - c**) shows the average across mice, while the lower row (**d - f**) shows the same average after normalizing each mouse's signal by min-max normalization. This normalization corrects for the different amplitudes in GCaMP signals across the different recording conditions, but preserves the features observed in each

recording condition. Note that the monoticity and non-monoticity of the responses in trials 1 and 2, respectively, are observed in each recording condition (**a - c**). **g** Normalized dopamine responses for all mice on trials 1 to 5. **h** Best fit by standard and with belief state reinforcement learning models. Data represents mean ± s.e.m.

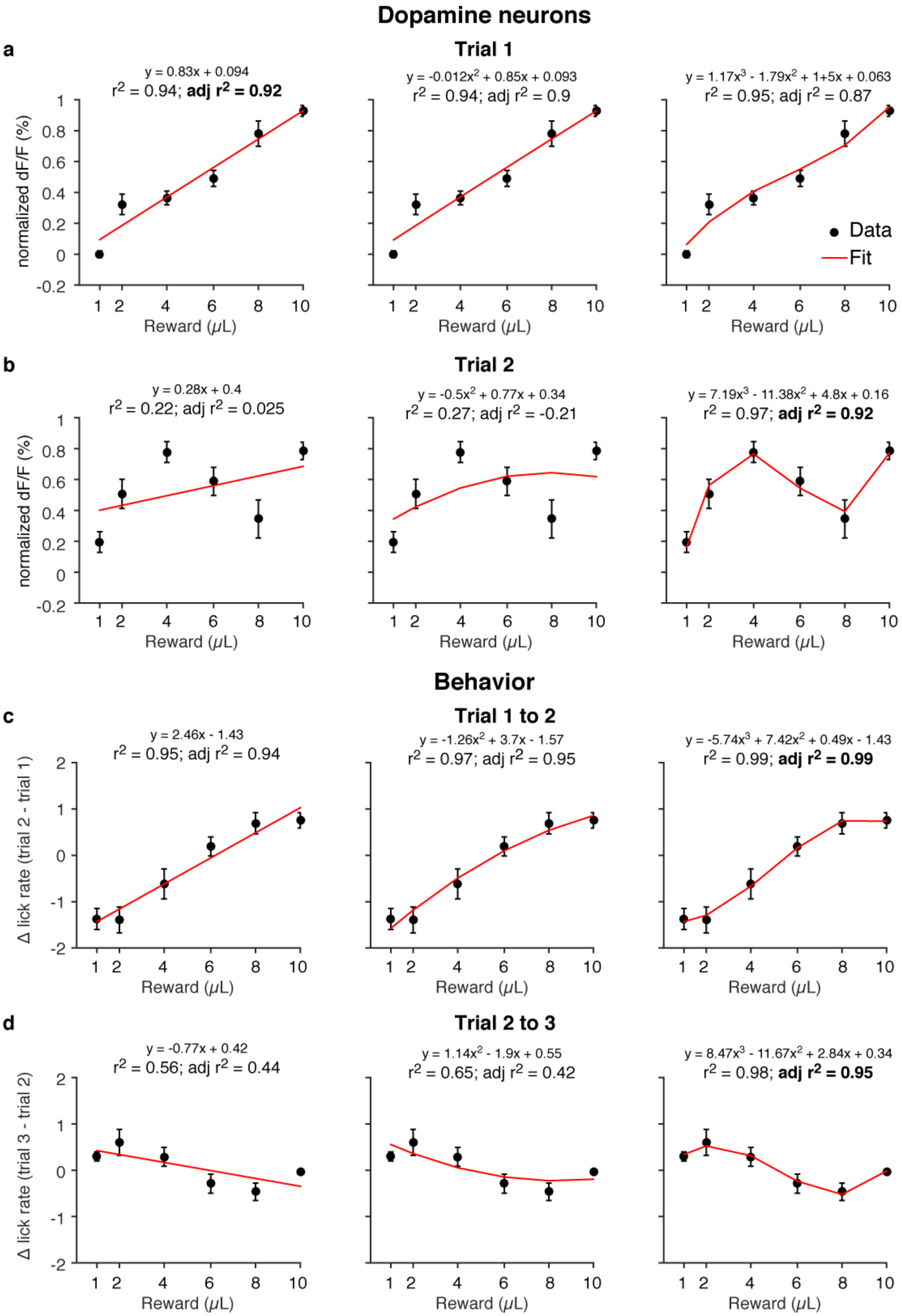
Dopamine neurons
a
Trial 1
y = 0.83x + 0.094
r2 = 0.94; adj r2 = 0.92
y = -0.012x2 + 0.85x + 0.093
r2 = 0.94; adj r2 = 0.9
y = 1.17x3 - 1.79x2 + 1+5x + 0.063
r2 = 0.95; adj r2 = 0.87
normalized dF/F (%)
Reward (μL)
Data
Fit
b
Trial 2
y = 0.28x + 0.4
r2 = 0.22; adj r2 = 0.025
y = -0.5x2 + 0.77x + 0.34
r2 = 0.27; adj r2 = -0.21
y = 7.19x3 - 11.38x2 + 4.8x + 0.16
r2 = 0.97; adj r2 = 0.92
normalized dF/F (%)
Reward (μL)
Behavior
c
Trial 1 to 2
y = 2.46x - 1.43
r2 = 0.95; adj r2 = 0.94
y = -1.26x2 + 3.7x - 1.57
r2 = 0.97; adj r2 = 0.95
y = -5.74x3 + 7.42x2 + 0.49x - 1.43
r2 = 0.99; adj r2 = 0.99
Δ lick rate (trial 2 - trial 1)
Reward (μL)
d
Trial 2 to 3
y = -0.77x + 0.42
r2 = 0.56; adj r2 = 0.44
y = 1.14x2 - 1.9x + 0.55
r2 = 0.65; adj r2 = 0.42
y = 8.47x3 - 11.67x2 + 2.84x + 0.34
r2 = 0.98; adj r2 = 0.95
Δ lick rate (trial 3 - trial 2)
Reward (μL)

Supplementary Figure 7. Polynomial fits to dopamine response and behavior for trials 1 and 2. Polynomials of degree 1 (left), 2 (middle) or 3 (right) were fit to the data and the corresponding r^2 and adjusted r^2, corrected for the degree of the polynomial, were computed. The highest adjusted r^2 is highlighted in bold. **a** Dopamine reward responses on trial 1 were best fit by a linear function. **b** Dopamine reward responses on trial 2 were best fit by a cubic function. **c** Change in anticipatory licking from trial 1 to trial 2 was best fit by a cubic function although the linear function also provided a good fit (r^2 = 0.94). **d** Change in anticipatory licking from trial 2 to trial 3 was best fit by the cubic function.

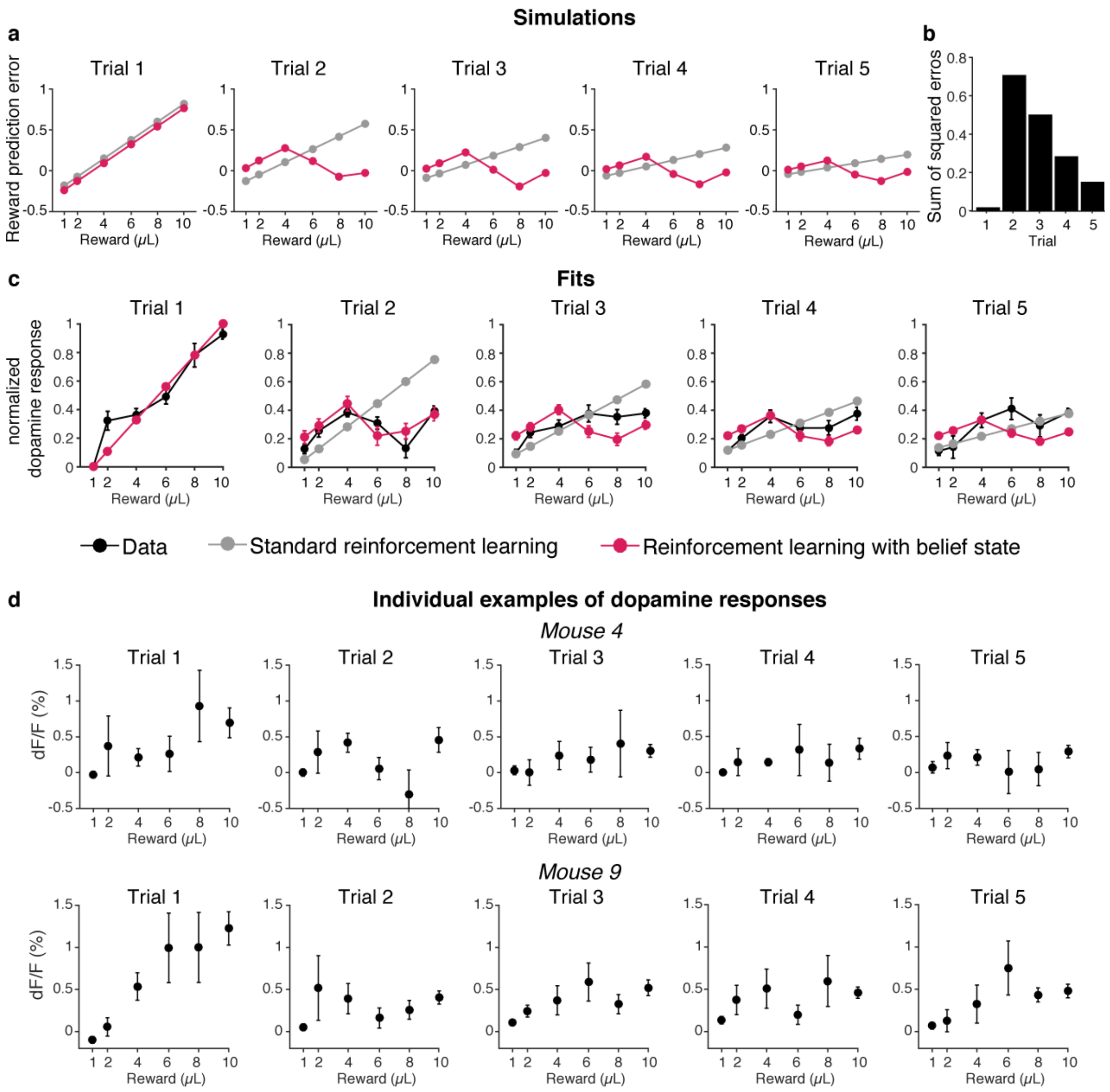

Supplementary Figure 8. Dopamine reward responses and model fits across trials. a Standard and belief state reinforcement learning models were simulated using the average parameters across mice (Supplementary Table 1). **b** Sum of squared errors between simulations from both models. Trial 2 shows the strongest difference. **c** Normalized dopamine responses to rewards and model fits to dopamine responses of the RL models without or with belief states, with two free initial values or priors. **d** Examples of individual dopamine responses. Data represents mean ± s.e.m.

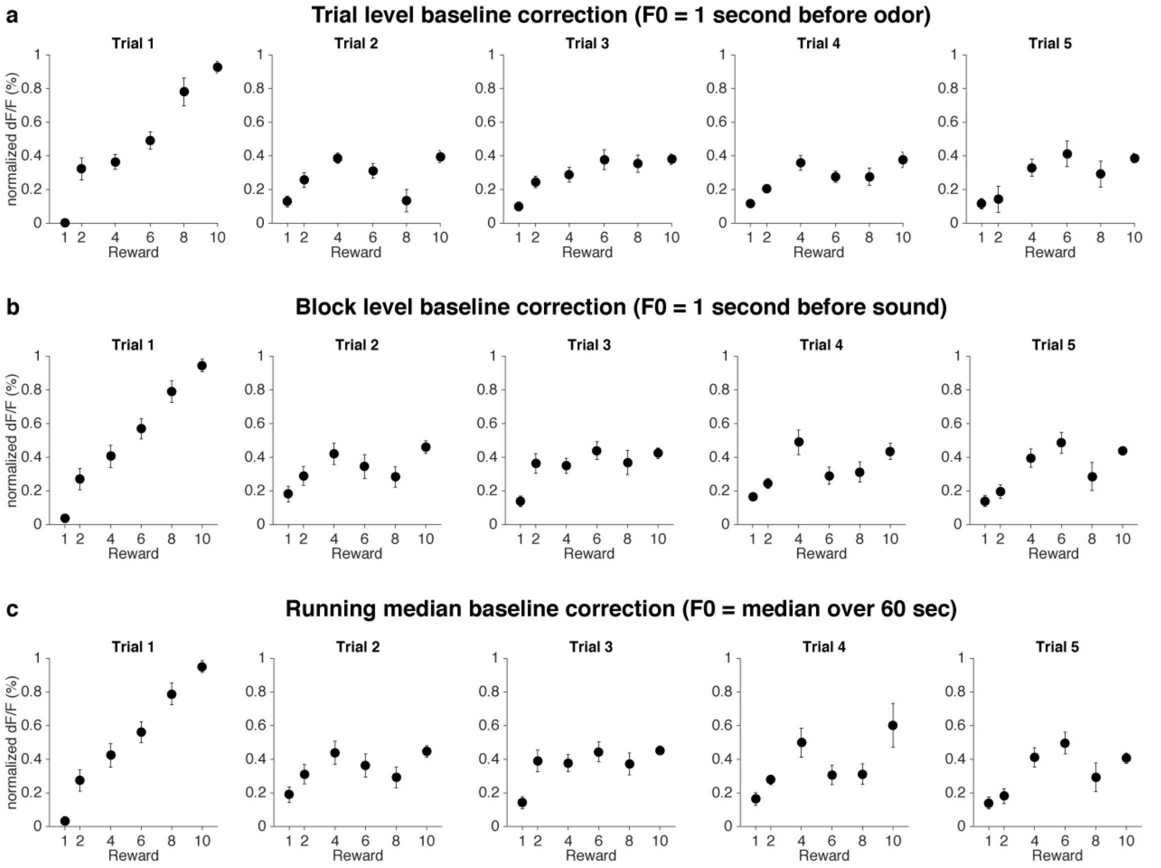

Supplementary Figure 9. Dopamine activity using different baseline correction methods. a Trial level baseline correction, using 1 second before odor onset as baseline. **b** Block level baseline correction, using 1 second before sound onset as baseline. **c** Running median baseline correction, using the median over a 60 second period centred on the current data point analysed as baseline. Data represents mean ± s.e.m. n = 11 mice

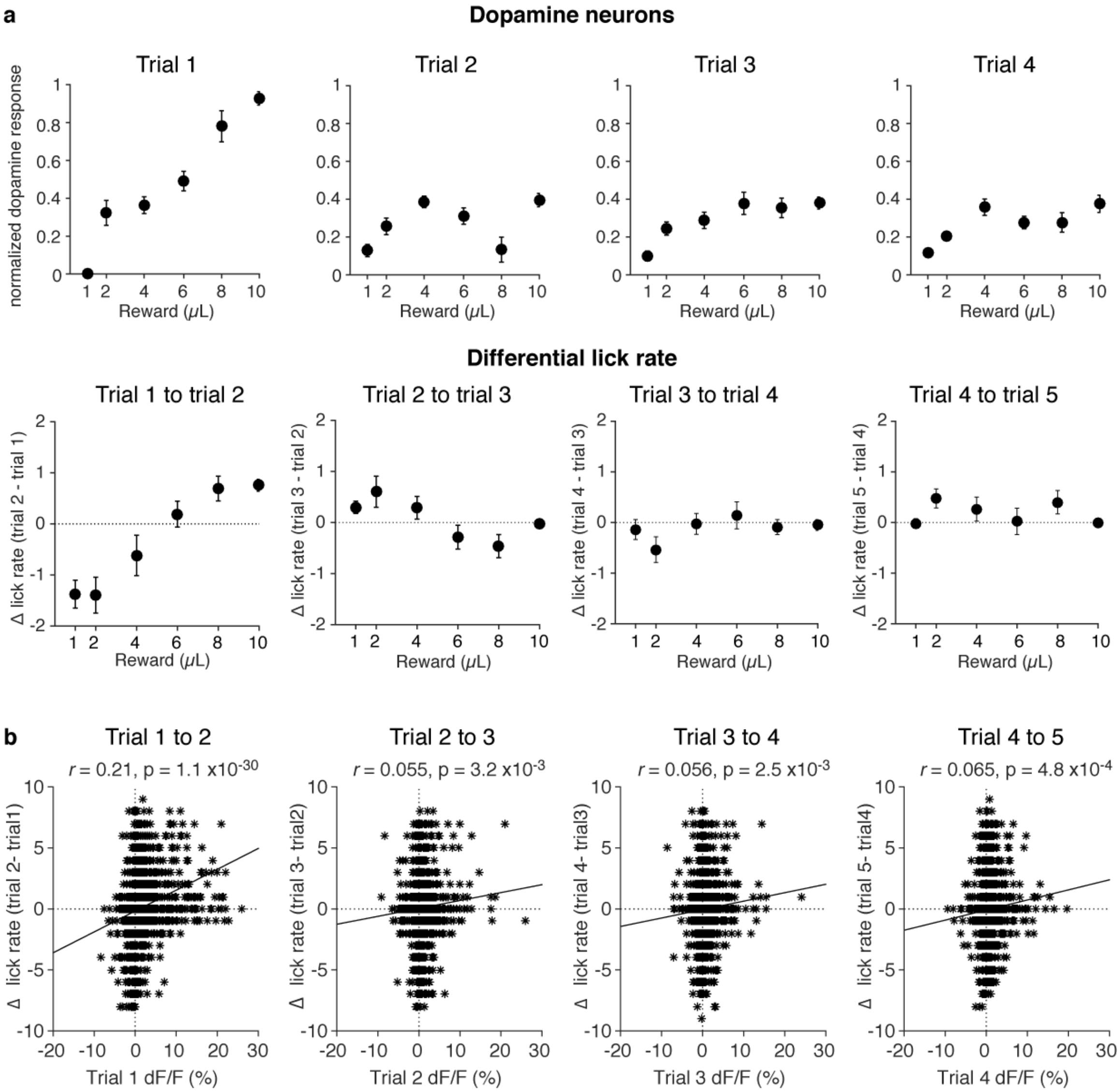

Supplementary Figure 10. Dopamine neuron activity and differential anticipatory licking within blocks. a Dopamine neuron activity on reward delivery for trials 2 to 5 (top) and corresponding differential lick rate (bottom). Data represent mean ± s.e.m. **b** Correlation analysis between dopamine neuron activity on trial t and lick rate change from trial t to trial t+1 within blocks. Each point represents an individual trial. n = 11

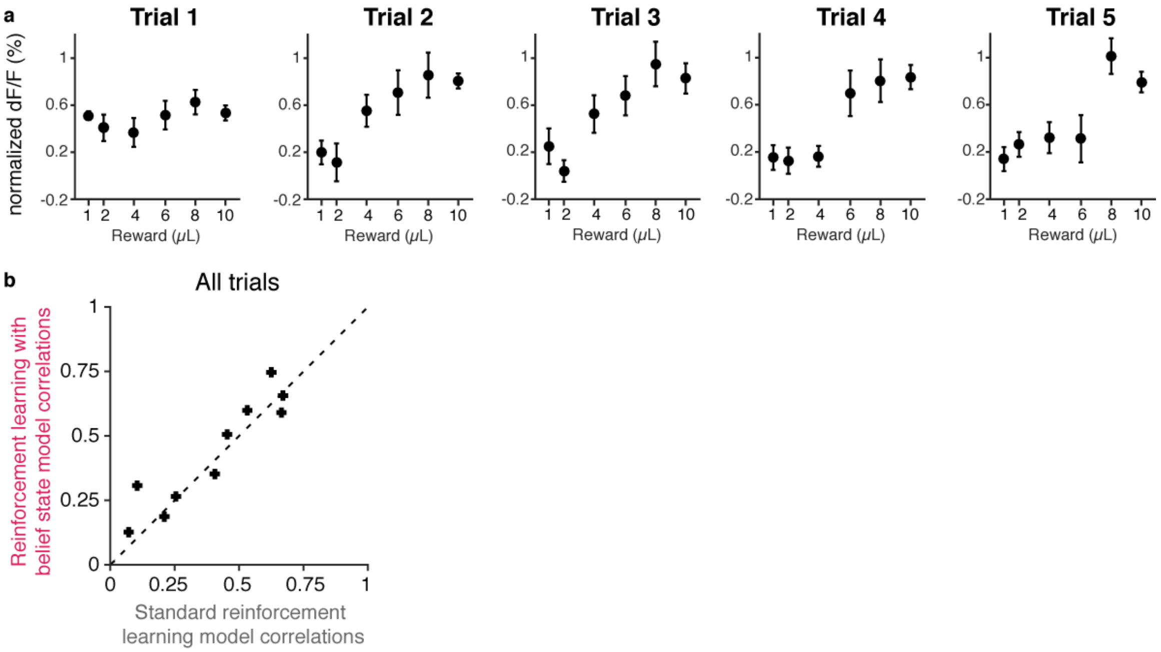

Supplementary Figure 11. Dopamine cue (CS) responses. a Normalized dopamine responses to odor presentation across trials. n = 11, data represents mean ± s.e.m. **b** Correlation between dopamine CS responses and estimated model values. The value functions from either model fits were positively correlated to the mice's anticipatory licking, but no model provided a better fit (signed rank test: $p = 0.32$).

Supplementary Tables

Model		Number of parameters	Parameters	Parameter estimates	Log-likelihood	BIC	Exceedance probability	Protected exceedance probability
Standard reinforcement learning	1 state, 1 fixed initial value (0.5)	1	learning rate (α)	0.257	-30.785	65.66	0.0809	0.1403
	1 state, 2 initial values depending on previous block (model in Fig. 3)	3	learning rate (α)	0.3	-24.755	61.79	0.2989	0.2073
			value following s_1 (V_1)	0.0077				
			value following s_2 (V_2)	0.357				
Reinforcement learning with belief state	2 states, 1 fixed initial prior (0.5)	2	learning rate (α)	0.0798	-33.452	75.09	0.0085	0.118
			sensory noise variance (σ)	0.425				
	2 states, 1 initial prior	3	learning rate (α)	0.279	-26.927	66.13	0.0214	0.122
			sensory noise variance (σ)	0.234				
			prior following s_1 (p_1) (with prior following s_2 p_2=1-p_1)	0.959				
	2 states, 2 initial priors depending on previous block (model in Fig. 3)	4	learning rate (α)	0.261	-22.174	**60.72**	**0.5112**	**0.2726**
			sensory noise variance (σ)	0.24				
			prior following s_1 (p_1)	0.989				
			prior following s_2 (p_2)	0.537				
	3 states, 2 initial priors depending on the previous block and 1 for the intermediate states	5	learning rate (α)	0.0829	-22.113	64.69	0.0791	0.1397
			sensory noise variance (σ)	0.166				
			prior following s_1 (p_1)	0.891				
			prior following s_2 (p_2)	0.0176				
			prior for intermediate rewards (p_3)	0.314				

Supplementary Table 1. Best-fitting parameter estimates shown as mean across mice and model comparison. Bayesian information criterion (BIC) and exceedance probabilities[3,4] both favoured the RL model with belief states with two initial free priors over other models. The best values are highlighted in bold.

Model		Number of parameters	Parameters	Parameter estimates	Log-likelihood	BIC	Exceedance probability	Protected exceedance probability
Standard reinforcement learning	1 state, 1 fixed initial value (0.5)	1	learning rate (α)	0.257	-57.4472	118.989	0.0379	0.1025
	1 state, 2 initial values depending on previous block (model in Fig. 3)	3	learning rate (α)	0.2915	-50.6742	113.631	0.3238	0.245
			value following s_1 (V_1)	0.0063				
			value following s_2 (V_2)	0.3383				
Reinforcement learning with belief state	2 states, 1 fixed initial prior (0.5)	2	learning rate (α)	0.0282	-59.8138	127.816	0.0064	0.0868
			sensory noise variance (σ)	0.4597				
	2 states, 1 initial prior	3	learning rate (α)	0.2711	-52.9573	118.197	0.0113	0.0893
			sensory noise variance (σ)	0.239				
			prior following s_1 (p_1) (with prior following s_2 $p_2=1-p_1$)	0.948				
	2 states, 2 initial priors depending on previous block (model in Fig. 3)	4	learning rate (α)	0.27	**-46.5757**	**109.529**	**0.5774**	**0.3713**
			sensory noise variance (σ)	0.315				
			prior following s_1 (p_1)	0.973				
			prior following s_2 (p_2)	0.556				
	3 states, 2 initial priors depending on the previous block and 1 for the intermediate states	5	learning rate (α)	0.046	-48.2943	117.06	0.0432	0.1051
			sensory noise variance (σ)	0.156				
			prior following s_1 (p_1)	0.934				
			prior following s_2 (p_2)	0.0026				
			prior for intermediate rewards (p_3)	0.389				

Supplementary Table 2. Best-fitting parameter estimates shown as mean across mice and model comparison on peak GCaMP response. Bayesian information criterion (BIC) and exceedance probabilities[3,4] both favoured the RL model with belief states with two initial free priors over other models. The best values are highlighted in bold.

Supplementary References

1. Kobayashi, S. & Schultz, W. Influence of Reward Delays on Responses of Dopamine Neurons. *J. Neurosci.* **28,** 7837–7846 (2008).
2. Fiorillo, C. D., Newsome, W. T. & Schultz, W. The temporal precision of reward prediction in dopamine neurons. *Nat. Neurosci.* **11,** 966–973 (2008).
3. Rigoux, L., Stephan, K. E., Friston, K. J. & Daunizeau, J. Bayesian model selection for group studies - Revisited. *Neuroimage* **84,** 971–985 (2014).
4. Stephan, K. E., Penny, W. D., Daunizeau, J., Moran, R. J. & Friston, K. J. Bayesian model selection for group studies. *Neuroimage* **46,** 1004–1017 (2009).
